# The characteristics of oviposition and hormonal and gene regulation of ovarian follicle development in Magang geese

**DOI:** 10.1186/1477-7827-11-65

**Published:** 2013-07-16

**Authors:** Qingming Qin, Aidong Sun, Rihong Guo, Mingming Lei, Shijia Ying, Zhendan Shi

**Affiliations:** 1 College of Animal Sciences, South China Agricultural University, Guangzhou 510642, China; 2 Institute of Animal Science, Jiangsu Academy of Agricultural Sciences, Nanjing 210014, China; 3 Institute of Food Safety and Monitoring Technology, Jiangsu Academy of Agricultural Sciences, Nanjing 210014, China

**Keywords:** Oviposition, Ovarian follice development, Gene expression, Hormone profile, Magang geese

## Abstract

**Background:**

Egg laying in Magang geese is characterized by extended interruption between clutches and lowing laying rate. Both the ovarian follicular development and ovulation characteristics, and the associated endocrine and molecular regulatory mechanisms involved are poorly understood, but could be important for guiding development of molecule aided selection of egg laying performances in geese. This study, therefore, recorded egg-laying characteristics of Magang geese, and the endocrine and molecular regulatory mechanisms of ovarian follicular development, maturation, and ovulation in Magang geese.

**Methods:**

Oviposition, ovarian follicle development, and reproductive hormone and gene expression profiles were observed in a small flock of Magang geese.

**Results:**

Greater than 73% of eggs were laid during the day. The average oviposition interval was 46.8 h (36–55 h). It took approximately 18 days for large white follicles to develop into mature F1 follicles; follicular growth was exponential. LHR expression levels increased from the small to the large mature follicles, but FSHR expression decreased in the granulosa and thecal layers. As the follicles matured, inhibin alpha and inhibin betaA expression increased in the granulosa layer. Activin IR, activin IIRA, activin IIRB, and beta-glycan expressions also increased as the follicles increased in size, but were more abundantly expressed in the thecal than in the granulosa layers. During the oviposition cycle, plasma concentrations of gonadal hormones decreased rapidly, whereas the level of PGFM peaked around ovulation. The profiles of activin, inhibin, follistatin, estradiol, and progesterone leading to ovulation were characterized.

**Conclusions:**

The molecular and endocrine mechanisms that regulate follicular development in Magang geese are similar to those in chickens. Moreover, gonadotropin regulation and interaction between activin, inhibin, and follistatin secretion may govern 3-stage maturation in the final preovulatory follicles in Magang geese. The rapid rebound of post-ovulatory secretions of inhibin and follistatin may inhibit recruitment of new SYF recruitment once a sequence of eggs is started, and may limit the egg clutch size to no more than the number of LYFs present before the first sequence egg.

## Background

Domestic geese are economically important for their ability to efficiently digest and utilize grass to produce meat, eggs, and feathers. The Magang goose is indigenous to Guangdong Province in southern China, with an egg production rate of 30 to 50 eggs per goose per annum. Total goose production is about 53 million annually, within a national total of 650 million or about 93% of the total goose production of the world [1]. The reproductive behaviors of Magang geese are characterized by strong seasonal egg-laying, strong incubation tendency, and a low egg-laying rate of no more than 40% [2,3]. These factors limit the annual laying capacity to approximately 35 eggs [4], with less than 30 goslings hatched. In contrast, prolific goose breeds have laying rates exceeding 60%, with at least 60 eggs laid in a single season [3,5]. Improving reproductive efficiency is an important goal for the Magang goose breeding program. Aside from eliminating incubation-prone individuals, one of the selection methods involves identifying frequent layers or geese with short oviposition intervals. This requires accurate recording of each oviposition by individual geese. Avian egg-laying is an event coordinated by hormones secreted by the pituitary gland and ovarian follicles [6,7] and by receptors for these factors on the surface of follicular cells [8]. Studying hormonal profiles during egg-laying cycles and gene expression patterns during follicular development will provide insights into the endocrine regulatory mechanisms of follicular maturation and ovulation and reveal the key roles of genes that determine oviposition cycle and egg-laying rate. Discovery of these genes is also important for molecule-aided selection of egg-laying performance [9]. The endocrine mechanisms that regulate the egg-laying cycle has been documented for the chickens [6,10,11], in which egg laying and ovarian follice recruitment are almost continuous, and so have the secretion patterns of progesterone and pgFM for graylag goose (*Anser anser*) [12]. However, for the Magang geese (*Anser cygnoid*) in which not only egg laying rate is low, but also the clutch or sequence egg size is limited to no more than the number of large yellow follicles (LYF) available before the first egg in a sequence, resulting extended interruption of over 20 days between the egg clutches [2]. The mechanisms regulating the follicle maturation, ovulation and oviposition have not been studied and are poorly understood. Therefore, this study aimed to characterize the egg-laying characteristics of Magang geese and to understand how particular endocrine and molecular factors influence ovarian follicular development and maturation that culminate in ovulation and egg-laying.

## Methods

### Animal experiments

#### Experiment 1: Egg-laying characteristics of free-range geese

Seventy laying geese were each fitted with an RFID (radio frequency identification) ring on the shank and were divided into 7 small flocks of 10 fowls each. Each flock was confined in a light-proof room (15 m^2^) and exposed to a short lighting program (11 L:13D). Each room was equipped with 2 nest boxes, each fitted with an RFID signal detector connected to a computer reader for individual identification. Video monitoring of laying behavior and the presence of eggs in the nest boxes helped determine the timing of oviposition; 527 ovipositions were recorded.

#### Experiment 2: Ovarian follicular development

On day 18 after termination of incubation, 4 non-incubating geese were selected; each was given an orally administered gelatin capsule containing 200 mg Sudan Black dye. A fat-soluble substance, Sudan Black is absorbed into the blood stream and readily deposited with yolk lipids onto the surface of each developing ovarian follicle, which includes large yellow follicles (LYF, with diameters > 9 mm), small yellow follicles (SYF, light yellow with diameters < 8 mm), and large white follicles (LWF, white follicles with diameters < 6 mm). The eggs were collected during the next 18 days and hard-boiled. The yolks were separated and transversely cut open to reveal the ring of Sudan Black. The ring diameter was measured with a pair of sliding calipers and used to calculate follicle volume and construct the follicle development curve.

#### Experiment 3: Blood sampling during the oviposition cycle

Blood samples were collected from 65 laying geese. To avoid causing excessive stress, which would influence normal oviposition, each bird was sampled only 3 times, each 72 h apart. Ten birds were sampled at each time point: 2, 4, 6, 12, 18, 24, 30, 34, 36, 38, 40, 42, 44, 45, 46, and 47 h after recorded oviposition, within the theoretical 48 h ± 2 h oviposition cycle. A denser sampling was arranged surrounding the oviposition and ovulation periods to allow accurate measurement of the variations in hormone concentration. All samples were collected from the wing vein using heparinized syringes. Plasma was separated within 3 h of collection, centrifuged at 2000 × *g* for 20 min at room temperature, and stored at −20°C.

#### Experiment 4: Collection of ovarian tissues

Using the RFID detection system and video camera monitoring, 10 Magang geese were selected and slaughtered 45 min after laying their first egg. Ovarian follicles (LYF, SYF, and LWF) were collected. The granulosa and thecal layers were separated from the 5 largest LYFs (F1 to F5); whole SYFs and LWFs were snap-frozen in liquid nitrogen and stored at −70°C. All experimental procedures were approved by South China Agricultural University Research Committee under the Animal Experimentation Code 2003.

### Hormone concentration measurements

Concentrations of estradiol (E2) and P4 were measured with medical diagnosis RIA kits (Beijing Northern Biotechnology Institute, Beijing, China). To minimize interference from steroid-binding proteins, 100 μL sample aliquots were mixed with an equal volume of 0.01 M PBS (pH7.4) and preheated in a 70°C water bath for 30 min to denature binding proteins and release bound hormone. The samples were analyzed according to the protocols supplied by the kit provider. The E2 kit had a sensitivity of 1 pg · mL^-1^, detection range of 1 pg · mL^-1^ to 160 pg · mL^-1^, and an intra-assay coefficient of variation (CV) of less than 10%; and those for P4 were 0.2 ng · mL^-1^, 0.5 ng · mL^-1^ to 10 ng · mL^-1^, and less than 10%, respectively.

Plasma PGFM (the metabolite of PGF_2α_), activin, inhibin A, and follistatin were measured using ELISA kits (R&D Systems China, Shanghai, China). The PGFM assay had a sensitivity of 1 pg · mL^-1^, detection range of 1 pg · mL^-1^ to 160 pg · mL^-1^, and intra-assay CV of less than 15%; those for activin were 0.2 ng · L^-1^, 0 ng · mL^-1^ to 40 ng · mL^-1^, and less than 10%; those for inhibin A were 1.0 pgmL^-1^, 0 pg · mL^-1^ to 2000 pg · mL^-1^, and less than 10%; and those for follistatin were 0.2 ng · mL^-1^, 0 ng · mL^-1^ to 8.0 ng · mL^-1^, and less than 10%. Serial dilutions of goose samples with high readings were used to generate binding patterns parallel to the standard curves, thus to validate the assays for measuring goose samples.

### Gene expression analysis

Real-time quantitative PCR was performed to quantify β-actin, LHR, FSHR, β-glycan, activin R, inhibin α, and inhibin βA mRNA expression in various tissues. Total RNA was extracted from the tissues using Trizol (Invitrogen), then reverse-transcribed to synthesize the first strand cDNA using a ReverTra Ace qPCR RT Kit (Toyobo, Osaka, Japan). PCR was performed in a 50 μL reaction volume using SYBR Green I Master Mix (Toyobo, Osaka, Japan) and 2.5 pmol primers (Table 1). An ABI PRISM_7500 sequence detection system (Applied Biosystems, Foster City, CA, USA) was used to determine the sequences of the reaction products. Upon completion of real-time Q-PCR, the threshold cycle (Ct, defined as the cycle at which a statistically significant increase in the magnitude of the signal generated by the PCR was first detected) values were calculated by the sequence detection software (SDS Version 1.2.2, Applied Biosystems). Expression levels were expressed as 2^-ΔCt^ (ΔCt = Ct_gene_ – Ct_β-actin_). All assays were performed in triplicate, and the amplified fragments were sequenced and verified through Blast searches against known chicken sequences in GenBank.

**Table 1:** Primers used in real-time quantitative PCR of genes in goose samples

**Gene name**	**Accession number**	**Prime sequences (5′ to 3′)**	**Annealing temperature (°C)**	**PCR product (bp)**
**LHR**	U31987.1	upstream: CTCTGTGATAACTTGCGTAT	52.8	**119**
		downstream: AAGGCATGACTGTGGAT	52.8	**279**
**FSHR**	NM_205079.1	upstream: CAACCTTCCCAAACTAC	52.8	**327**
		downstream: ATTTCCTGAATCCCATT		
**ß-Glycan**	M77809.1	upstream: GTGAACTTCCCAATAGCA		
		downstream: AGTGTCCAGACCGTAGAAC		
**Activin IIRα**	D31899.1	upstream: ACTGACTTCCTCAAGGCTAA	52.8	**131**
		downstream: GAGATGGCGGGTTTATGT	55.8	**114**
**Inhibin α**	NM_001031257.1	upstream: GGGCTGGAAGAGGTAGGTGAAGAT	55.8	**113**
		downstream: GGAGGACACGTCGCAGGTGAT		
**Inhibin ßA**	NM_205206.1	upstream: CAGTGGACACCCGAAAGA		
		downstream: GCACAGGTCACAGGCAAT		
**Activin IR**	NM_204560.1	upstream: TGCCTGGATGGTTTCGTC	55.8	**189**
		downstream: GCCATTTCTCATCGGGACT		
**Activin IIRβ**	NM_204317.1	upstream: ATTTACTACAACGCCAACT	55.8	**143**
		downstream: CAGCCTTTCTTCACCAG		
**β-Actin**	L08165.1	upstream: GAGAGAAATTGTGCGTGA	55.8/52.5	**195**
		downstream: TCCATACCCAAGAAAGAT		

### Statistical analysis

The egg laying pattern during a day was calculated as the percentage of ovipositions detected at each hour of the day over the total number of ovipositions in Experiment 1. Laying interval was calculated as the mean time between 2 adjacent ovipositions in a lay-incubation cycle in Experiment 2. Differences in PGFM, E2, P4, activin, inhibin A, and follistatin concentrations at each laying time were analyzed by one-way analysis of variance, and the means were compared by the Tukey-Kramer method with each time point as a treatment level. Gene expression was analyzed with each follicle class as a treatment level. All values are expressed as mean ± SEM. All statistical analyses were performed with SAS software Version 8.01 (SAS Institute Inc. Cary, NC. USA).

## Results

### Egg-laying characteristics

**Figure 1:**
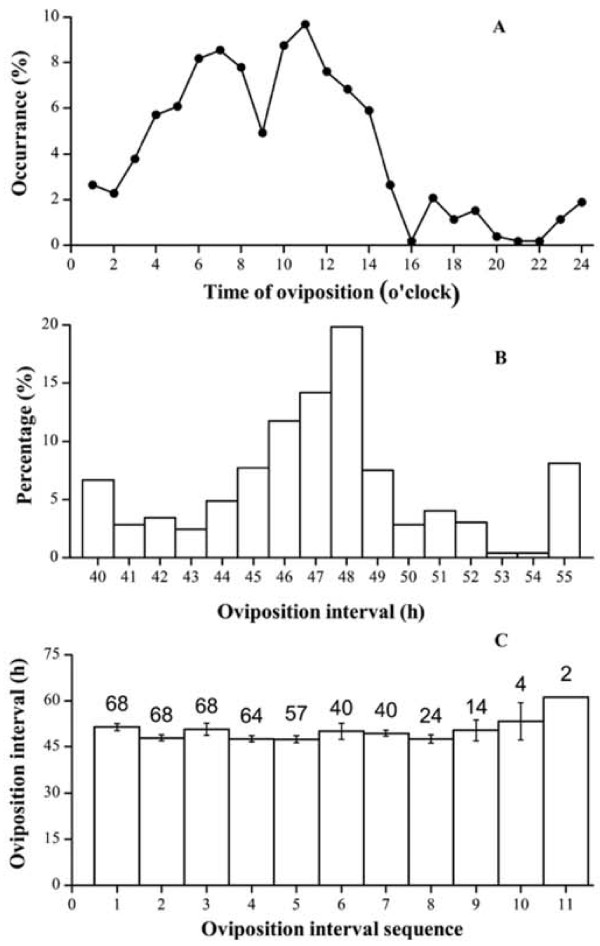
**Oviposition characteristics of Magang geese: Daily egg-laying pattern (A), oviposition interval distribution (B) and relationship between oviposition interval and egg sequence (summarized from 527 ovipositions).** The number atop the bar in graph **C** depicts the number of observations.

Figure 1A depicts the time distribution of eggs laid during a day, calculated from 527 eggs laid by 69 geese during one laying–incubation cycle. The majority of eggs were laid during the day, comprising 73.1% of all eggs laid. Only 26.9% of ovipositions occurred at night (18:00 pm to 6:00 am). The brief drop in oviposition around 9:00 am was associated with the lights coming on at 8:00 am (Figure 1A), which caused nesting geese to leave the nests for feed and water.

The average oviposition interval, calculated from recordings of 498 ovipositions by 69 geese, was 46.8 ± 1.4 h, varying from 37 h to 55 h. Approximately 10% of the intervals were exceptionally longer, greater than 55 h, possibly due to the stress caused by catching and collection of blood samples (Figure 1B). Along the laying sequence, which lasted from 4 to 12 eggs between geese, oviposition intervals varied close to 48 h until the 8^th^ interval or before the 9^th^ oviposition. Thereafter, the interval tended to lengthen towards the end of laying and the start of incubation behavior (Figure 1C).

### Follicular development speed

**Figure 2:**
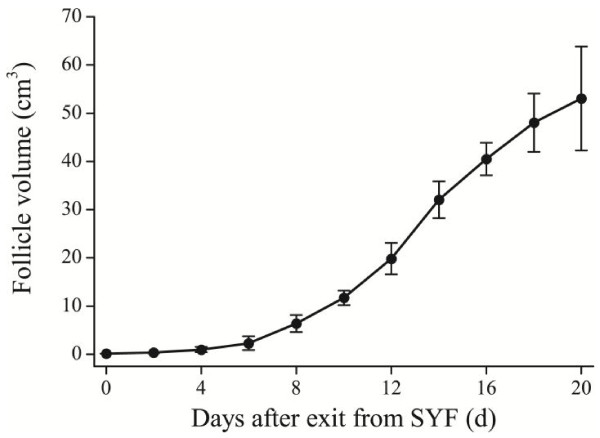
**Ovarian follicular development curve constructed from diameter of Sudan Black dye circumference yolk of laid eggs from 4 Magang geese.** Vertical bars represent the standard error of the mean.

After oral feeding with Sudan Black dye, the eggs carried a black ring that occurred on the surface but moved gradually into the yolk center as egg-laying proceeded. The black ring persisted in the yolk center 16 to 18 days after dye feeding, suggesting it took approximately 16 days for LWFs to develop into mature F1 LYFs, which took another 2 days to be laid as an egg after ovulation. The yolk volume inside the ring, calculated from the ring circumference and diameter of each size class of follicle, was used to construct a follicle growth curve (Figure 2). Follicle growth was most rapid from F6 to F2. Both absolute and relative expansions from F2 to F1 slowed, possibly because the follicles were maturing and reached their limit. This curve was best fitted by a 4-parameter logistic equation of V = −0.663 + 58.231/(1 + 72.130 * exp(−0.371*D)) (R^2^ = 99.9%), in which V stands for the volume of the ovarian follicle and D for the number of days from the small yellow follicle stage.

### Plasma hormone concentrations

**Figure 3:**
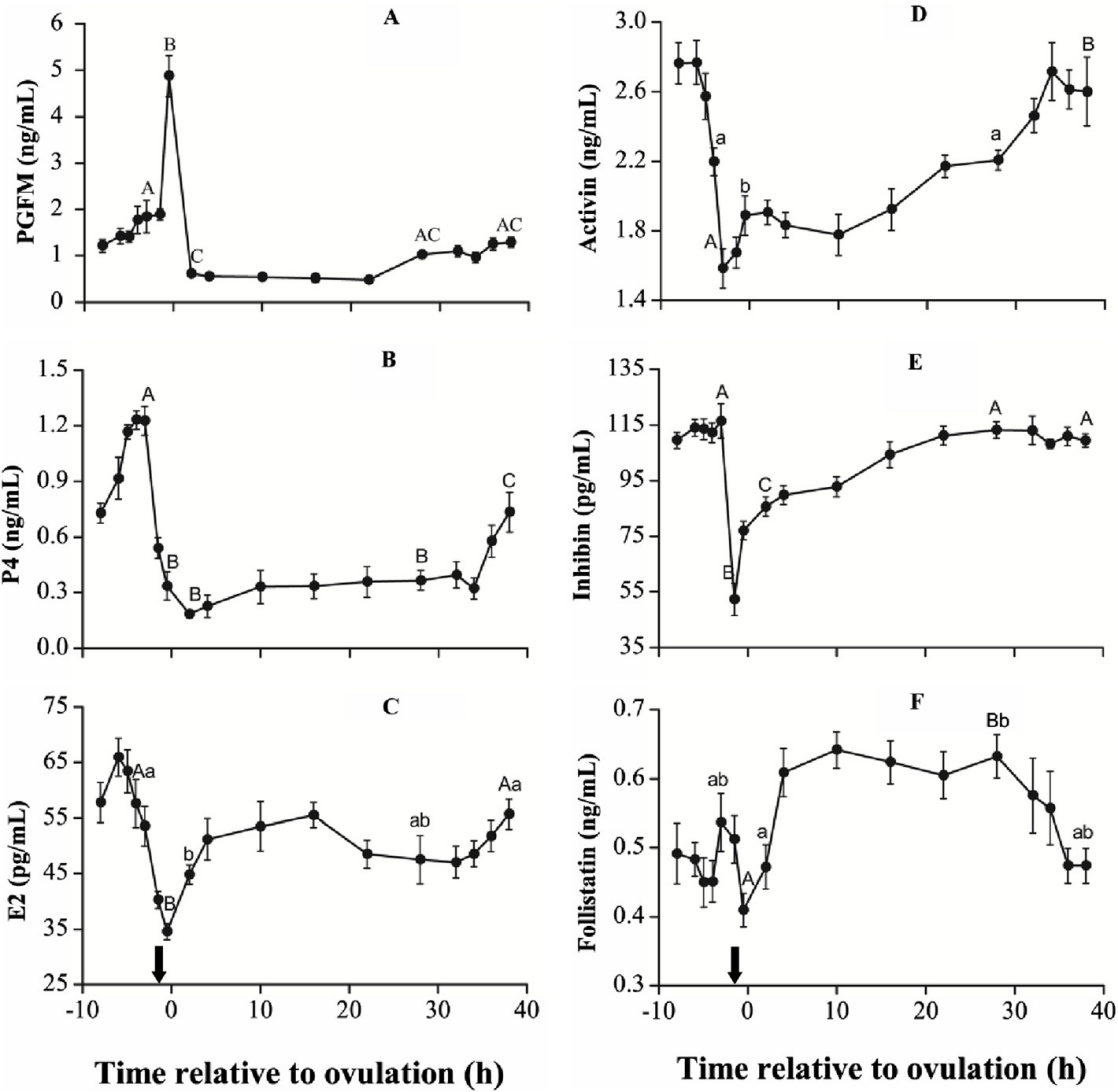
**Plasma concentrations of PGFM (A), P4 (B), E2 (C), activin A (D), inhibin (E) and follistatin (F) during the oviposition or ovulation cycle.** Vertical bars represent the standard errors of the means. The arrow depicts the time of oviposition. Values marked with different letters were significantly different (a–b: *P* < 0.05; A–B, A–C, B-C: *P* < 0.01).

Within the 48-h blood sampling period, the most significant change in plasma pgFM was a single abrupt peak that corresponded to oviposition. From the intermediate levels of 2 pg/mL at 1.5 h before oviposition, plasma PGFM increased abruptly to a 5 pg/mL peak 0.5 h before oviposition. After 2 h, the concentration returned to basal levels (Figure 3A). The concentrations remained low until 28 h, at which point they rose slightly to form a second marginal increase from 4 h to 1.5 h before the next oviposition.

Plasma P4 concentrations remained at basal levels during most of the cycle, and then started to increase steadily approximately 10 h before oviposition. The increase was stable for approximately 6 h until the peak (approximately 1.2 ng/mL) at 3 h to 4 h before oviposition. The concentrations abruptly decreased to a nadir (~0.2 ng/mL) immediately after oviposition (Figure 3B).

Within the egg-laying cycle, plasma E2 concentrations reached their nadir immediately before oviposition, then rebounded to intermediate levels within 5 h. This level was maintained for another 30 h before increasing further and peaking over 4 h, or about 6 h to 5 h before the next oviposition (Figure 3C).

The cycle trough of activin A was detected 3 h before oviposition, earlier than for E2 and P4 (Figure 3D). Within 3 h, activin A transiently increased to approximately 1.80 ng/mL until 10 h after oviposition. A second increase started thereafter, and steadily rose to the peak level at approximately 34 h post-oviposition, or 14 h before the next oviposition. The peak lasted for 8 h and started to decrease 6 h before the next oviposition. Thus, the prelaying plasma activin A peak occurred earlier and lasted much longer than those of E2 and P4.

Changes in plasma inhibin were marked by a sudden fall 1.5 h prior to oviposition (Figure 3E). The concentrations immediately rose again after oviposition, reaching a plateau within 16 h that lasted until 3 h before the next oviposition.

Plasma follistatin exhibited a two-phase variation during oviposition, with intermediate fluctuations 10 h before oviposition and a high plateau 5 h to 30 h post-oviposition (Figure 3F).

### Gene expression

**Figure 4:**
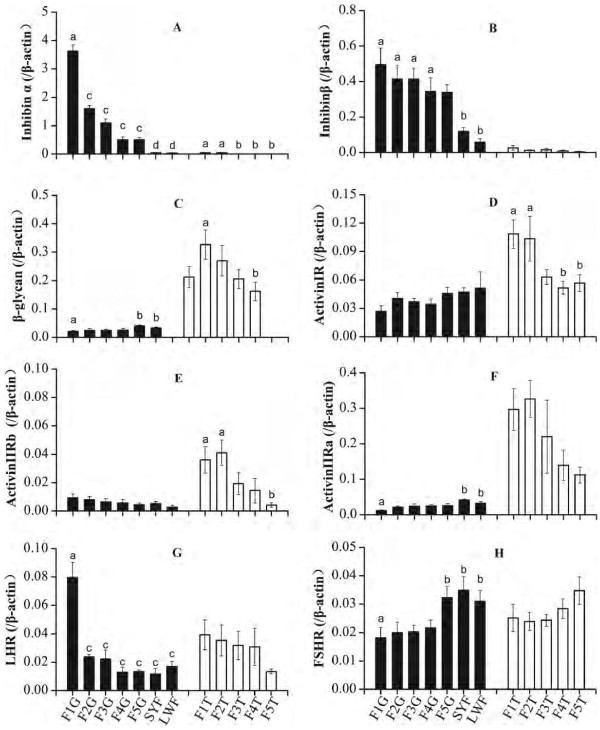
**mRNA expression levels relative to β-actin of inhibin α (A), inhibin βA (B), β-glycan (C), activin RI (D), activin RIIA (E), activin RIIB (F), LHR (G) and FSHR (H), in the granulosa (G) and thecal (T) layer of various size ovarian follicles in Magang geese.** Fn represents the hierarchical order of follicle size, with the largest follicle designated as F1. Vertical bars represent standard errors of the mean. Means not marked by a common letter are significantly different (a-b, b-c, c-d: P < 0.05; a-c, a-d: P < 0.01).

Among the 8 genes pertinent to follicular development, expression of inhibin alpha, inhibin βA, and the 3 activin R subunits were higher in the granulosa than in the thecal layer. Expression of inhibin α and inhibin βA increased progressively from the small to the large follicles, particularly for inhibin alpha (Figure 4A). In the theca, inhibin βA was expressed at low levels and without variation among the differently sized LYF (Figure 4B). Expression of the inhibin receptor β-glycan was much higher in the theca than in the granulosa layer. The levels increased in the theca of the smallest F5 until F2, and then decreased in F1 (Figure 4C). In contrast, β-glycan expression decreased in the granulosa layer from the LWFs and SYFs, to the more mature LYFs. As for the activin receptors, activin IR (Figure 4D), activin IIRα (Figure 4E), and activin IIRβ (Figure 4F), expression was higher in the thecal layer than in the granulosa layer. Furthermore, the changes in expression of these receptors in the theca were similar to those of β-glycan: increasing with LYF maturity. In the granulosa layer, expression of activin IR and activin IIRα decreased from the small to the large follicles similar to β-glycan, except for activin IIRβ, which increased. The expression of LHR gradually increased in the granulosa and thecal layers of LWFs, SYFs, and the more mature preovulatory LYFs, with the expression levels most strikingly enhanced in the F1 granulosa layer (Figure 4G). Conversely, FSHR expression was inversely proportional to that of LHR in the different class of follicles. In particular, FSHR expression decreased from high levels in the LYFs, SWFs, and F5 follicles to low levels in the larger LYFs (Figure 4H).

## Discussion

We recorded the egg-laying patterns of Magang geese, oviposition time distribution during the day, and oviposition intervals or cycle lengths. In addition, we measured the expression of reproductive hormones throughout the cycle, and the expression of genes involved in hormonal regulation in the context of ovarian follice development in order to understand the regulatory mechanisms of egg-laying in Magang geese. The results of this study constitute the first thorough investigation of oviposition characteristics and regulation in geese.

Most oviposition occurs during the day and scarcely at night in Magang geese. This is somewhat different from European graylag geese (*Anser anser*) reported by Çelebi and Güven [12], which lay eggs mostly during the early part of the day. Oviposition is thought to be regulated by an interaction of endogenous rhythms with daily photoperiod [6], which determines neuroendocrine regulation of ovulation. It appears that Magang geese differ from European geese in the photo-entrainment of oviposition. In this regard, Magang geese are similar to the chicken which also lays eggs during the day, but unlike the duck, a waterfowl that lays eggs at night or before photophase.

The oviposition interval of Magang geese, being 46.8 h on average, is consistent with previously reported observations [2,12]. This means egg-laying occurs every other day, and will make the daily laying rate close to 50%. This laying rate is rarely observed under practical production conditions, as the actual laying rate seldom reaches 30% [3], possibly due to variability between individual birds and the presence of non-laying birds in the flock, as observed by Yang et al. [4]. In this study, some geese laid only very few eggs (4 to 5 eggs), while some laid up to 12 eggs before establishing incubation behavior. These latter good layers also had oviposition intervals stably close to 48 h, compared with the rather irregular intervals that could last up to 80 to 90 h in some poor laying geese. These phenomena are quite different from the short intervals, as short as 36 h, in some prolific geese that had peak laying rate exceeding 60% [3]. Therefore, in selection program for Magang geese, the target should be set for selecting high number of eggs and also stable oviposition intervals. In addition, the oviposition interval became increasingly longer towards the end of the clutch. Lengthening of the oviposition interval at the end of the clutch might result from the lowering of LH secretion caused by rising secretion of PRL [2], which would slow follicle maturation.

Follice developmental speed was recorded by marking follicle diameter with Sudan Black dye. This not only unraveled the time course required for SYFs to develop to ovulation, 18 days, but also helped to construct a mathematical model of follicle growth that was hitherto unknown. The 18 days for SYFs to develop to ovulation could be established by laying of the previous 8 to 10 eggs in a clutch, but it is an accurate reflection of natural conditions. It took between 20 to 25 days from the end of incubation to laying a new clutch of eggs in Magang geese [2]. In other words, the time required for LWFs to develop to ovulatory maturity is 20 or so days on average. In addition, during practical production, the laying-incubation cycle in Magang geese is approximately 50 days [2]. This consists of the 7 to 10 days for terminating incubation behavior, the 20 to 25 days required for initiation of laying, and the 18 to 20 days required to lay the whole clutch of eggs.

During the stages of egg-laying, ovarian follicles of different sizes are exposed to the same endocrine milieu, which affects each individual follicle through the expression of receptors on the cell surface. The factors involved in ovarian follicular development and ovulation include pituitary gonadotropins [11] and the autocrine/paracrine factors secreted by the follicles themselves [8,13]. We characterized the expression profiles of these factors in this study. The dramatic upregulation of LHR and inhibin alpha subunit, in the largest F1 follicle, and also changing patterns of other genes in granulosa and theca layers, were all similar in Magang goose to those reported for chicken [14-17]. These results indicate that the progression of follicular development and the molecular mechanisms involved, i.e., the endocrine regulation by gonadotropin and autocrine/paracrine regulation by inhibin/activinR, are much similar for geese and chickens even though the former have lower laying rates than the latter [2].

Apart from the aforementioned differences in chickens, geese have much longer oviposition cycles. This could be due to the maturation process and hormonal profile, which affects the oviposition cycle. In this study, the concentrations of 6 hormones or metabolite were measured within a single ovulation cycle. We found that the changing hormonal patterns were consistent with those previously reported for chickens [6,8,10] and Graylag goose [12]. Among the hormones, PGF_2α_ plays a pivotal role in oviposition cycle regulation in avian species, such that the enhanced synthesis and release of uterotonic PGF_2α_ stimulates uterine muscle contraction, which culminates in expulsion of the egg [12,18]. The blood concentrations of PGFM, the metabolic molecular form of PGF_2α_, are normally measured to reflect changes in PGF_2α_ secretions [12,18]. In this study, the single peak in plasma PGFM was detected during the narrow oviposition time window, similar to previous results in chickens and geese [12,18]. PGF_2α_ is synthesized in uterine and follicle tissues and its concentration peaks can be detected in the blood of chickens independent of oviposition [16]. Therefore, analyzing the expression and structure of the genes associated with PGF_2α_ synthesis and their association with oviposition cycle length and egg-laying rate in geese are of particular interest.

For progesterone, another hormone instrumental to ovulation, the plasma concentrations during the oviposition cycle were comparable to those of Magang geese (*Anser cygnoids*) under laying state [2], but much lower than those reported for Graylag geese (*Anser anser*) [12]. These differences may result from interspecies differences in progesterone secretion or from differences in assay methods. This issue needs to be clarified in future studies. Nevertheless, the interoviposition progesterone variation pattern in Magang geese strikingly resembles that reported for Graylag geese [12], with the preovulatory P4 increasing at 14 h and peaking 3 h before oviposition. Considering the interval between the preovulatory P4 peak and ovulation is about 3.5 hours in both geese and chickens [6,10,11], even though the goose ovulation cycle is about 22 h longer than in chickens, goose F1 follicles clearly take longer to mature. In other words, follicular development and maturation immediately after ovulation occurs at a slower pace in geese. This may be a factor in the prolonged oviposition cycle of geese and may reduce egg-laying frequency and rate.

Immediate to ovulation, both plasma concentrations of activin and inhibin decreased dramatically. This indicated the mature F1 follicle also secrets large amount of activin in Magang geese. This phenomen is contrary to situation in the chicken that activin was considered to be mainly secreted by less developed F4 to F3 follicles [8]. Also, the residual inhibin concentration nearing 50 pg/ml was about half the preovulatory peak of 110 pg/ml, indicating the F2 follicle also secreted copious amount of inhibin, rather than the small amount in the chicken model [8]. Ready secretion of inhibin by F2 was also seen by the rapid rebound of plasma concentration in less than 5 hr after the ovulation, also followed by follistatin concentration rebounds. Compared with inhibin secretion rebound at 10 hr post-ovulation in chicken hens whose ovipositin interval was only 24 hr or so and new SYF recruitment occurs daily [11], post-ovulatory inhibin secretion in Magang geese was recovered highly rapid. Moreover, these infer that, during laying a sequence or clutch of eggs, copious amount of these hormones are always present in blood circulation. Since inhibin and follistatin counteract the activin’s role of promoting small follicle development [8], the continued presence of inhibin and follistatin in blood may inhibit new SYF recruitment into preovultory LYF development after Magang geese entering into lay [2], causing clutch egg size limited to no more than the number of LYFs present before laying of the first egg in a sequence. This also explains taking place of the near 20-day interruption between two clutches of eggs as discussed above, and also in some non-incubating geese [3].

Within 10 h to 15 h after ovulation, the hormonal profile is characterized by the static secretion of progesterone, transient secretions of activin and inhibin, and robust secretions of estradiol and follistatin. Activin A enhances granulosa cell proliferation and mediates expression of FSHR and LHR [8,13]. The increase in follistatin secretion during the first 12 after ovulation should counteract the effects of low transient activin A secretions to delay follicular maturation during this stage and to inhibit progesterone secretion. Nevertheless, increasing gonadotropin secretions following ovulation of the F1 follicle and subsequent withdrawal of negative feedback [6,19] may stimulate the remaining F2 follicle and enhance estradiol secretion.

From mid-cycle onwards, i.e., 15 h after ovulation, the plasma activin A concentrations continued to increase with inhibin concentrations (though at a lower magnitude), whereas follistatin concentrations remained stable. These changes in hormonal balance or increase in “activin tone” may regulate the second stage of follicular development or maturation, i.e., functional upgrading of the F2 follicle to F1.

As the F1 follicle continues to mature, plasma activin A concentrations continue to increase, peaking at 34 h after ovulation, or 14 h before the next ovulation. This activin A peak occurred concomitantly with the fall of follistatin concentrations, but with preovulatory increases in P4 and estradiol. Considering the plasma inhibin concentrations remain stable during this stage, the “activin tone” is undoubtedly strengthened, facilitating final maturation of the F1 follicle that ultimately triggers the preovulatory LH surge and ovulation. The earlier decrease in plasma E2 concentrations compared with P4 before ovulation is also consistent with the phenomenon observed in chickens, wherein the more mature F1 follicle secretes less E2 [20]. This indicates that as the preovulatory F1 follicle matures, gonadal steroidogenesis shifts from E2 to P4.

## Competing interests

The authors declare that they have no competing interests.

## Authors’ contributions

QMQ, ADS, RHG, MML, SJY and ZDS devised the study and participated in its design. QMQ did the practical analysis, advised by RHG. QMQ and RHG sampled the material. ZDS and MML wrote the manuscript. MML, SJY and ZDS corrected the manuscript. All authors read and approved the final manuscript.
